# p21^WAF1/CIP1^ Upregulation through the Stress Granule-Associated Protein CUGBP1 Confers Resistance to Bortezomib-Mediated Apoptosis

**DOI:** 10.1371/journal.pone.0020254

**Published:** 2011-05-26

**Authors:** Cristina Gareau, Marie-Josée Fournier, Christine Filion, Laetitia Coudert, David Martel, Yves Labelle, Rachid Mazroui

**Affiliations:** Department of Molecular Biology, Medical Biochemistry, and Pathology, Faculty of Medicine, Laval University, CHUQ Research Centre/St-François d'Assise Research Centre (CRCHUQ/CRSFA), Quebec, Canada; St. Georges University of London, United Kingdom

## Abstract

**Background:**

p21^WAF1/CIP1^ is a well known cyclin-dependent kinase inhibitor induced by various stress stimuli. Depending on the stress applied, p21 upregulation can either promote apoptosis or prevent against apoptotic injury. The stress-mediated induction of p21 involves not only its transcriptional activation but also its posttranscriptional regulation, mainly through stabilization of p21 mRNA levels. We have previously reported that the proteasome inhibitor MG132 induces the stabilization of p21 mRNA, which correlates with the formation of cytoplasmic RNA stress granules. The mechanism underlying p21 mRNA stabilization, however, remains unknown.

**Methodology/Principal Findings:**

We identified the stress granules component CUGBP1 as a factor required for p21 mRNA stabilization following treatment with bortezomib ( =  PS-341/Velcade). This peptide boronate inhibitor of the 26S proteasome is very efficient for the treatment of myelomas and other hematological tumors. However, solid tumors are sometimes refractory to bortezomib treatment. We found that depleting CUGBP1 in cancer cells prevents bortezomib-mediated p21 upregulation. FISH experiments combined to mRNA stability assays show that this effect is largely due to a mistargeting of p21 mRNA in stress granules leading to its degradation. Altering the expression of p21 itself, either by depleting CUGBP1 or p21, promotes bortezomib-mediated apoptosis.

**Conclusions/Significance:**

We propose that one key mechanism by which apoptosis is inhibited upon treatment with chemotherapeutic drugs might involve upregulation of the p21 protein through CUGBP1.

## Introduction

The proteasome is a large multi-subunit complex responsible for the degradation of various proteins, including cell cycle regulators and apoptotic factors, via both ubiquitin-dependent and –independent mechanisms [1], [2]. Incubation of proliferating cells with proteasome inhibitors induces apoptosis [3]–[6]. The proteasome inhibitor bortezomib has recently been approved for clinical use, showing strong antitumor activity in multiple myeloma and other hematological tumors [7], [8]. However, solid tumors of different organ origins are refractory to bortezomib, and this resistance is also observed in cancer cell lines derived from solid tumors [9]–[12]. The mechanisms by which cancer cells resist to bortezomib are still largely unknown, although it is postulated that this resistance might involve the activation of a general stress response [9]–[13].

We have recently reported the formation of stress granules following treatment with bortezomib [14]. Stress granules (SG) are cytoplasmic bodies whose formation is induced by various types of stress such as ionizing radiations [15], hypoxia [16], viral infection [17], [18], and proteasome inhibitors [14], [19], [20]. Since such stress stimuli are known to inhibit translation initiation, it was speculated that SG represent sites where the translation of specific mRNAs is repressed [21]–[24]. SG might repress translation in part by limiting the interaction of mRNAs with ribosomes [21], [22]. The potential role of SG in translation repression is supported by several reports showing that specific mRNAs are inefficiently repressed when RNA-binding proteins, which contribute to SG formation, are altered [15], [18], [20], [25], [26]. These foci also contain small ribosomal subunits, translation initiation factors and signaling molecules [21], [27]. In accordance with the proposed role of SG as storage sites for untranslated mRNAs, these foci are devoid of large ribosomal subunits [28] . Once the stress is relieved SG gradually disassemble, leading to the recovery of translation required for cell survival. It is thus postulated that SG formation is central to the stress response, which reprograms gene expression towards the synthesis of proteins that are essential to allow cells to cope with stress and thus survive [21]. Indeed, the induction of SG upon exposure to hypoxia [29], oxidative stress (e.g. arsenite) [19], or bortezomib treatment [14] leads to resistance of tumor cells to apoptosis. One mechanism underlying such resistance appears to involve the sequestration and inactivation of pro-apoptotic factors such as RACK1 and TRAF2 in SG [29]–[31]. Other mechanisms by which SG may antagonize apoptosis could involve the sequestration of mRNA encoding key anti-apoptotic factors within SG, thereby preventing their degradation. Although such mechanisms have not been formally demonstrated, evidence nevertheless exists suggesting a role for SG in the regulation of mRNAs encoding anti-apoptotic proteins [15], [20], [32].

We and others have previously suggested a potential role of SG in the regulation of one such antiapoptotic factor, namely the expression of p21*^WAF1/CIP1^* mRNA [20], [32]. p21 is a well-known cyclin-dependent kinase inhibitor induced following various stress stimuli, including proteasome inhibitors. As a proliferation inhibitor, p21 was suggested to play a role in tumor suppression by promoting cell cycle arrest or by inducing apoptosis [33]–[36]. Conversely, other laboratories reported that stress-induced upregulation of p21 can protect tumor cells from apoptosis [37]–[41]. Whether p21 upregulation promotes resistance to apoptosis mediated by proteasome inhibitors in cancer cells is currently unknown. It is well established that stress-mediated upregulation of p21 involves transcriptional mechanisms. Other studies indicate that p21 expression is also regulated post-transcriptionally, mainly at the level of mRNA stability, in response to various SG-inducing stress stimuli such as UV radiation [42], [43] and oxidative stress [32]. We have previously shown that the proteasome inhibitor MG132 induces the sequestration of p21 mRNA into SG, which correlates with its stabilization [20].

Due to its short half-life, p21 mRNA is expressed at very low levels under normal growth conditions [20], [42], [43]. We showed that treatment of HeLa cells for 3 to 6 h with proteasome inhibitors such as MG132 or lactacystin induces both SG formation and p21 mRNA accumulation, due in part to stabilization of the latter [20].

CUGBP1 belongs to the CELF family of RNA binding proteins and is aberrantly expressed in myotonic dystrophy type I [44]–[46]. CUGBP1 controls mRNA splicing in the nucleus. In the cytoplasm, CUGBP1 was shown to promote either mRNA decay [44], [47]–[52] or translation of its associated mRNAs [44]–[46], [53]. The translational function of CUGBP1 has been investigated in normal cells. These studies showed that CUGBP1 increases p21 translation in myocytes, thereby leading to their differentiation [46], [53], and in aged normal fibroblasts, where it results in a senescent-like phenotype [54]. The role of CUGBP1 in apoptosis was unsuspected until recently. Kress and coll. generated mice in which the *Cugbp1* gene was inactivated by homologous recombination [55] . Although *Cugbp1^−/−^* mice are viable, they display significant perinatal mortality and growth retardation. Moreover, germ cells derived from *Cugbp1^−/−^* mice are more prone to apoptosis than wild-type cells [55]. Recent studies have shown that CUGBP1 accumulates into SG following arsenite treatment, indicating that CUGBP1 might play a role in cellular stress response [32], [56].

In the study herein, we identified CUGBP1 as a factor required for p21 mRNA stabilization upon bortezomib treatment. We show here that CUGBP1 accumulates into SG following bortezomib treatment, together with its associated p21 mRNA. Upon depletion of CUGBP1, bortezomib-mediated p21 mRNA localization in SG and therefore its stabilization are lost, thereby leading to its misexpression. Altering the expression of p21 either by depleting CUGBP1 or p21 itself promotes bortezomib-mediated apoptosis. Our study unveils p21 as a potential therapeutic target.

## Results

### Bortezomib induces p21 mRNA accumulation followed by its translation

**Figure 1:**
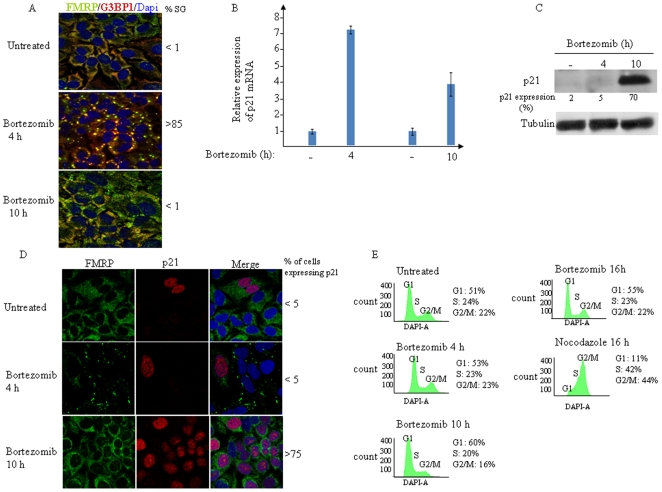
Bortezomib upregulates p21 mRNA expression. (A) HeLa cells were treated with 2 µM bortezomib for 4 h or 10 h, fixed, permeabilized, and then processed for immunofluorescence using antibodies against different SG markers. DAPI is used as a nuclear stain. Pictures were taken using 63X objective. The percentage of cells harboring SG (>3 granules/cell) from five different fields and three different experiments containing a total of 1000 cells is indicated. (B) qRT-PCR of p21 mRNA. Following treatment with bortezomib for the indicated period, cells were collected and total RNA content was then isolated. The amount of p21 was quantified by real time-PCR relative to GAPDH mRNA using the ΔΔCt method. Results are expressed as the mean ± SEM (error bars) of triplicate measurements. (C) HeLa cells were treated with 2 µM bortezomib for the indicated time, and the level of p21 was analyzed by western blotting using specific antibodies (*top* panel). Tubulin is used as a loading standard. The amount of p21 was determined by quantitation of the signals on films by densitometry using Adobe Photoshop software and expressed as a percentage relative to the amounts of tubulin. The results are representative of five different experiments. (D) HeLa cells were treated as in (A). p21 protein was detected using anti-p21 antibodies and SG were visualized with anti-FMRP antibodies. (E) Bortezomib does not affect cell cycle at the time points analyzed. Cells were treated with 2 µM bortezomib or with 0.8 µg/ml nocodazole for the indicated interval, collected, washed with PBS, and then fixed with 3% paraformaldehyde for 20 min. Cells were washed with PBS, stained with DAPI (1 µg/mL) and analyzed by flow cytometry.

We have previously shown that bortezomib induces the reversible formation of SG [14]. We therefore analyzed the expression of p21 during the assembly and disassembly of SG following bortezomib addition. In HeLa cells, SG formation, which occurred 4 h after bortezomib treatment (Fig. 1A; *center* panel) correlated with a drastic increase in the steady-state level of p21 mRNA (Fig. 1B). Time course analysis of SG formation shows that the percentage of cells forming SG remains high between 4 and 7 h of bortezomib treatment. (Fig. S1A). At those stages, however, p21 mRNA was not being efficiently translated (Fig. 1C and Fig. S1B), despite its sustained accumulation (Fig. S1C). Prolonged treatment with bortezomib (10 h) resulted in complete SG disassembly (Fig. 1A; *bottom* panel, and Fig. S1A) and concomitant efficient p21 protein production (Fig. 1C and Fig. S1B), despite some reduction (30–40%) in p21 mRNA level (Fig. 1B). We obtained similar results using two other cell lines, namely the MCF-7 human breast carcinoma and Calu-I human lung carcinoma cells (Fig. S2A–B). We next used immunofluorescence to corroborate our results of p21 expression. We found that p21 was below the detection threshold in untreated cells or upon treatment with bortezomib for 4 h (Fig. 1D; *top* and *center* panels, respectively, and Fig. S1A) to 7 h (Fig. S1A). Very few (<5%) untreated cells or cells treated with bortezomib for 4–7 h did express p21, however, which might either reflect a stressed state or normal cell cycle arrest in these cells. However, when bortezomib treatment was extended to 10 h, p21 was highly expressed and accumulated in the nucleus, concomitantly with SG disassembly (Fig. 1D; *bottom* panel; see also Fig. S1A). To ensure that the proteasome is still inhibited at the time when SG disassembled, we monitored the expression of a reporter GFP fused to a proteasome-targeting sequence (GFPu). In the absence of any proteasome inhibitors, the GFPu protein is constitutively degraded by the proteasome [57], [58]. Treatment of these GFPu-expressing cells with bortezomib prevented the degradation of GFPu protein, which accumulated. This accumulation of GFPu persists even after prolonged treatment with bortezomib for 10 h and beyond (Fig. S3A and data not shown), indicating that the activity of the proteasome was not recovered. Moreover, prolonged treatment (10 h and beyond) with bortezomib resulted also in the accumulation of proteasome substrates such as p27 [59]–[61] (Fig. S3B and data not shown) and p53 [59]–[61] (data not shown). These results indicate that the disassembly of SG occurred without reactivation of the proteasome. Nuclear p21 has previously been shown to promote cell cycle arrest, mainly by interfering with cyclin E/cdk2 activity [62]. On the other hand, other studies have reported that nuclear p21 accumulation promotes the formation of cyclin D-cdk4 complexes in cancer cells, thus contributing to increased proliferation and reduced apoptosis in tumors [63]. Therefore, we investigated whether nuclear p21 accumulation which occurred concomitantly with SG disassembly (cf. 10 h following bortezomib treatment; Fig. 1D, S1A, and S1D) might reflect cell cycle arrest. Time course-FACS analysis of cell cycle progression shows that bortezomib treatment does not affect cell cycle (Fig. 1E). As a control for cell cycle arrest we used nocodazole [64], which as shown in Figure 1E stops cellular progression at G2/M. p21 was also shown to inhibit apoptosis through inactivation of pro-apoptotic proteins such as apoptosis signal-regulating kinase 1 (ASK1) and caspase-3 in the cytoplasm [40], [65], [66]. We found that protracted treatment with bortezomib (16 h) induces a significant accumulation of p21 in the cytoplasm (Fig. S1A; *bottom-right* panel; see also Fig. S1E), which may reflect a role of this protein in antagonizing bortezomib-mediated apoptosis (see below). Overall, the results thus far show that bortezomib-mediated p21 upregulation involves two steps: (i) the initial accumulation of p21 mRNA, which correlates with SG formation, and (ii) the secondary translation of p21 mRNA, which correlates with SG disassembly. This pattern of events suggests that SG might serve as foci for the accumulation of p21 mRNA in a translationally repressed form.

### Bortezomib treatment induces p21 mRNA segregation with SG and its stabilization

**Figure 2:**
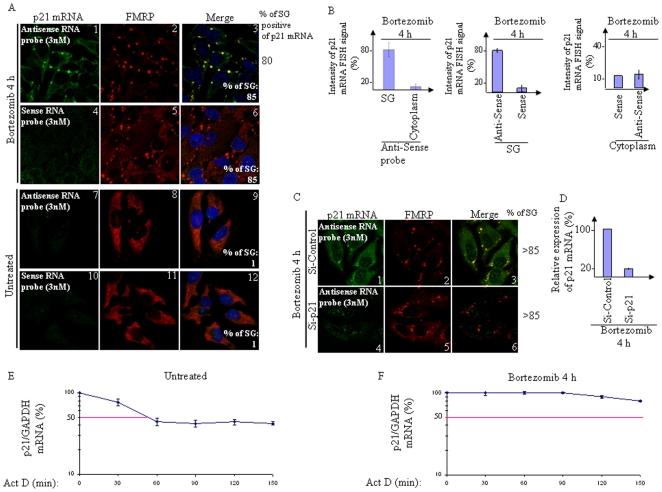
The accumulation of p21 mRNA following bortezomib treatment involves its stabilization and correlates with its segregation in SG. (A–B) Bortezomib induces p21 mRNA segregation into SG. Following treatment with bortezomib (2 µM) for 4 h, HeLa cells were fixed, permeabilized, and then incubated with 3 nM of an Alexa Fluor 488-labeled antisense RNA probe to detect p21 mRNA (panels 1–3 and 7–9) or with the Alexa Fluor 488-labeled sense probe as control (panels 4–6 and 10–12). SG were detected using anti-FMRP antibodies. The percentage of SG (>3 granules/cell) under each condition is indicated at the bottom of the right panels. The percentage of cells harboring SG positive for p21 mRNA is indicated at the right. Shown are typical results from five different fields and three different experiments containing a total of 1000 cells. (B) Densitometry quantification of FISH signal with Adobe Photoshop software. The number of pixels and mean intensities were recorded for the selected regions (SG, cytoplasm and background) using Photoshop. The mean intensity was multiplied by the number of pixels for the region selected in order to obtain the absolute intensity. The absolute intensity of the background region was subtracted from each region of interest. In order to compare the intensity between two given regions of interest, relative intensities were next calculated. Relative intensities correspond to the absolute intensities normalized according to the absolute intensity of the region of reference. (C–D) p21 mRNA FISH signal is lost upon p21 knockdown. Cells were treated with p21 or control siRNA. Seventy-two hours later, cells were treated with bortezomib (2 µM for 4 h) in order to induce SG, and then processed for FISH to detect p21 mRNA coupled with immunofluorescence in order to visualize SG (C). In (D), cells were lysed and their RNA content analyzed for the expression levels of p21 mRNA using qRT-PCR. p21 mRNA levels were standardized against the non-target GAPDH mRNA and expressed as a percentage of the initial mRNA levels (i.e. at time zero) present at each time point. The error bars correspond to the SD of three independent experiments. (E–F) Bortezomib induces p21 mRNA stabilization. HeLa cells were left untreated (E) or were treated with bortezomib (2 µM) for 4 h (F). Cells were then incubated with 5 µg/ml actinomycin D for 30 minutes and collected at the indicated time points following the incubation. Total RNA was isolated and the level of p21 mRNA expression was determined by qRT-PCR, standardized against GAPDH mRNA, and expressed as a percentage of the initial mRNA levels (i.e. at time zero) present at each time point. The error bars correspond to the SD of three independent experiments.

We next assessed whether accumulating p21 mRNA was localized in bortezomib-induced SG. For that purpose, we used fluorescent *in situ* hybridization (FISH) to visualize p21 mRNA in fixed HeLa cells, using fragile X mental retardation protein (FMRP) [14], [67], [68] as a marker for staining SG (Fig. 2A). Resulting staining reflected a typical localization of FMRP in the cytoplasm in control experiments (Fig. 2A, panels 8 and 11), and in SG upon bortezomib treatment (Fig. 2A, panels 2 and 5). FISH showed a barely detectable p21 mRNA staining in untreated cells (Fig. 2A, panel 7; see also Fig. S4A and S4C), in keeping with the low amount of p21 mRNA and protein observed under normal growth conditions (cf. Fig. 1B–C and S1C). Bortezomib induced the localization of p21 mRNA in SG in a high percentage (∼80%) of cells, as assessed by FISH using the targeted (antisense) probe (Fig. 2A, panels 1 and 3; see also Fig. S4A–B). Quantification of the intensity of p21 mRNA FISH signal in SG compared to the cytoplasm revealed that the major fraction (∼80%) of p21 mRNA was associated with SG (Fig. 2B; *left* panel; see also Fig. S4B). Background FISH signal was detected in SG and in the cytoplasm using the untargeted (sense) probe (Fig. 2A, panels 4 and 6, and Fig. 2B; *center* and right panels). Similar background signal was detected in the cytoplasm of bortezomib-treated cells using the antisense probe (Fig. 2A, panels 1 and 3; see also the right panel of Fig. 2B for quantification), as compared to the signal obtained using the sense probe (Fig. 2A, panels 4 and 6, and the right panel of Fig. 2B). The SG-linked FISH signal which is detected using antisense probe was specific to p21 mRNA since it was lost in cells treated with p21-targeted siRNA to deplete the transcript (Fig. 2C; compare panels 1 and 4, and Fig. 2D). p21 depletion it self did not affect SG formation, as suggested by the localization of several SG markers (Fig. 2C, and data not shown) ruling out the possibility that the loss of SG-linked FISH signal observed in p21-depleted cells was due to a general cellular failure to form SG. As expected, prolonged treatment (10 h) of cells with bortezomib induces SG disassembly and p21 mRNA localization to the cytoplasm and to the nucleus (Fig. S4A and S4C; *bottom-left* panels). At this time, however, the overall p21 mRNA FISH signal is weaker than the one observed in cells treated with bortezomib for 4 h (Fig. S4A; compare *bottom-left* with *center-left* panel), in keeping with the decreased amount of p21 mRNA as measured by qRT-PCR following SG disassembly (Fig. 1B). Overall, the results (Fig. 2A–B and S4A–B) show that p21 mRNA is quantitatively recruited to bortezomib-induced SG. We then assessed the possible contribution of p21 mRNA localization in SG in its accumulation. We have previously reported that the formation of bortezomib-induced SG requires heme-regulated inhibitor kinase (HRI) [14]. Thus, we examined whether altering SG formation via HRI depletion affects p21 mRNA accumulation. We found that HRI depletion indeed prevented p21 mRNA accumulation following bortezomib treatment, as assessed by qRT-PCR (Fig. S5A). HRI was efficiently depleted as assessed by western blotting (Fig. S5B), and as expected, its depletion completely prevented SG formation (Fig. S5C). Altogether, these results show that bortezomib treatment induces p21 mRNA accumulation in SG. We then addressed the hypothesis that this p21 mRNA accumulation reflects its stabilization. For this purpose, we determined the effect of bortezomib on p21 mRNA stability in the presence of actinomycin D. Upon inhibition of *de novo* RNA synthesis with actinomycin D, the relative half-life of p21 mRNA was increased from 60 min ±10 min in untreated cells to >3 h upon bortezomib treatment (Fig. 2E–F), reflecting bortezomib-induced accumulation of p21 mRNA (Fig. 1B). As shown in Figure 2E however, the p21 mRNA decay included a plateau phase. This indicated that the p21 mRNA decay may not be a first order process. This type of decay occurs if two or more degradation processes with different rate constants are involved. Subpopulation of p21 mRNAs may be restricted to discrete subcellular locations that prevent their decay. Also, the decay of some p21 mRNAs may be mediated by competing pathways and/or decay mechanisms utilizing distinct trans-acting complexes, which could then affect their decay rates. Nevertheless, our results suggest that p21 mRNA segregation in SG contributes to its stabilization and accumulation upon bortezomib treatment. Next we sought to determine which factor is involved in p21 mRNA localization in SG.

### Role of CUGBP1 in p21 mRNA accumulation and subsequent translation during bortezomib treatment

**Figure 3:**
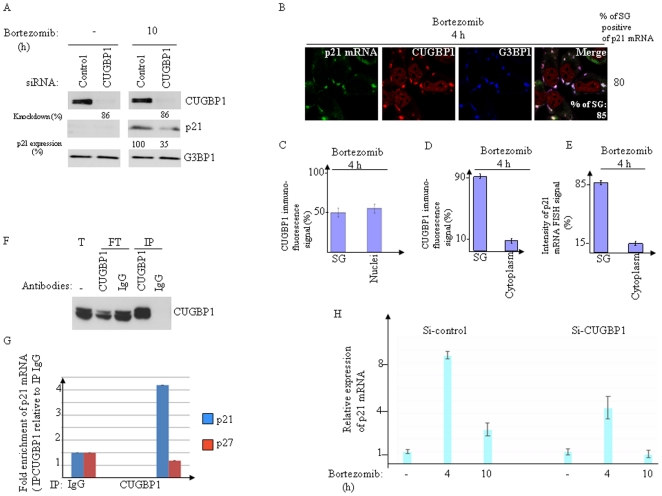
CUGBP1 binds to p21 mRNA and promotes its accumulation and expression upon bortezomib treatment. (A–D) Seventy-two hours following transfection with CUGBP1, or control siRNAs, HeLa cells were treated with bortezomib (2 µM) for either 10 h (A) or 4 h (B). (A) Protein extracts were prepared and analyzed by western blot to detect CUGBP1, p21, and G3BP1 proteins (loading standards) using the appropriate antibodies. The percentages of CUGBP1 knockdown and p21 expression were determined by quantitation of the signal on films by densitometry using Adobe Photoshop as described in Figure 1C. Shown are typical results of three experiments. (B–D) p21 mRNA is quantitatively recruited into SG where it co-localizes with CUGBP1. (B) Cells were processed for FISH to detect p21 mRNA coupled to immunofluorescence to visualize SG using antibodies against CUGBP1 and G3BP1. The percentage of SG (>3 granules/cell) is indicated at the bottom of the right panel. The percentage of cells harboring SG positives for p21 mRNA is also indicated on the right of the figure. Shown are typical results from five different fields and three different experiments containing a total of more than 1000 cells. (C–D) Densitometry quantification of CUGBP1 immunofluorescence signal in SG versus nuclei (C) and in SG versus the cytoplasm (D) using Adobe Photoshop software as described in Figure 1. (E) Densitometry quantification of p21 mRNA FISH signal was done as described in Figure 2. (F–G) HeLa cells were treated with bortezomib for 4 h and their extracts were used to immunoprecipitate CUGBP1 with anti-CUGBP1 antibodies and with IgG as a control. IP: Immunoprecipitate; FT: flow-through following immunoprecipitation; Total: the input used for immunoprecipitation. (F) Proteins were analyzed by western blot for CUGBP1 immunoprecipiation. (G) mRNAs were isolated from each immunoprecipitate and quantified by qRT-PCR. The amounts of p21 mRNA and p27 mRNA (as control) were normalized against GAPDH mRNA. (H) HeLa cells were treated with CUGBP1-specific siRNA, or with control (non-specific) siRNA as described above, incubated with 2 µM bortezomib for either 4 or 10 h, lysed, and their total RNA isolated and analyzed for p21 mRNA levels by qRT-PCR. The amount of p21 mRNA was normalized against that of GAPDH mRNA as described in Figure 2.

**Figure 4 pone-0020254-g004:**
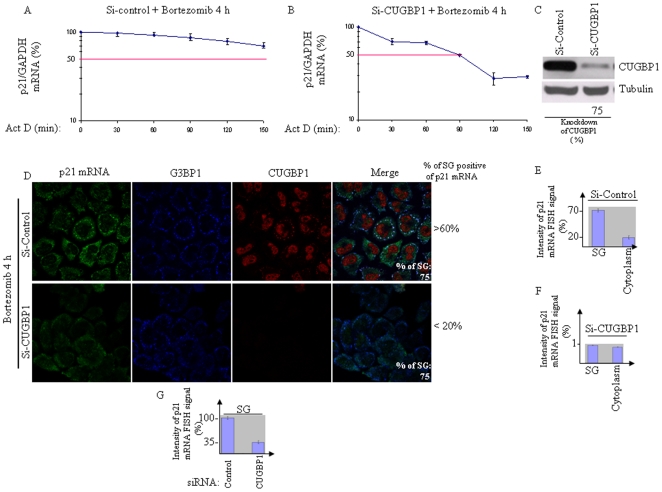
Reducing CUGBP1 levels prevents bortezomib-mediated p21 mRNA stabilization and accumulation in SG. (A–B) HeLa cells were treated with non-specific (A), or with CUGBP1-directed siRNA (B), incubated with bortezomib (2 µM) for 4 h, and actinomycin D (5 µg/ml) was then added for a period of 30 minutes. Cells were collected at the indicated time points following the incubation and total RNA was isolated. The level of p21 mRNA expression was measured by qRT-PCR, standardized against GAPDH mRNA, and expressed as a percentage of the initial mRNA levels (i.e. at time zero) present at each time point. The error bars correspond to the SD of three independent experiments. (C) Depletion of CUGBP1 was assessed by western blot using anti-CUGBP1 antibodies and quantified as described in Figure 3. (D) HeLa cells were treated with CUGBP1-specific siRNA, or with control siRNA as described above, treated with bortezomib (2 µM) for 4 h, and then fixed, permeabilized, and incubated with 3 nM of an Alexa Fluor 488-labeled antisense RNA probe to detect p21 mRNA. SG were detected using anti-G3BP1 antibodies. Depletion of CUGBP1 was assessed by immunofluorescence using anti-CUGBP1 antibodies. The percentage of SG is indicated at the bottom of the right panels. The percentage of cells harboring SG positives for p21 mRNA is also indicated. Those percentages are representative of typical results from three different experiments counting more than 1000 cells. (E–F) Densitometry quantification of p21 mRNA FISH signal in SG versus the cytoplasm in mock-depleted cells (E) and CUGBP1-depleted cells (F) as described in Figure 1. (G) Densitometry quantification of p21 mRNA FISH signal in SG in mock-depleted versus CUGBP1-depleted cells as described in Figure 2.

The SG components HuR and CUGBP1 have been shown to bind p21 mRNA, thereby regulating its expression [14], [54], [69]. While HuR promotes p21 mRNA stabilization through interaction with its AU-rich element in the 3′-untranslated region, CUGBP1 interacts with the GC-rich element in the 5′ region of p21 mRNA coding sequence and promotes its translation in senescent fibroblast cells. We investigated the role of these two proteins in the upregulation of p21^WAF/CIP1^ observed upon proteasome inhibition in HeLa cells. We found that depletion of HuR had no effect or rather slightly increased p21 protein expression following bortezomib treatment (data not shown). Depletion of CUGBP1 using two specific siRNAs had no effect on p21 protein expression under normal growth conditions but prevented bortezomib-mediated p21^WAF/CIP1^ upregulation as assessed by western blot analysis using anti-p21 antibodies (Fig. 3A and data not shown). These results indicate that CUGBP1 promotes p21 upregulation upon proteasome inhibition. As a first step to investigate how CUGBP1 up-regulates p21 upon proteasome inhibition, we performed co- localization studies between CUGBP1 and p21 mRNA. Under normal growth conditions, CUGBP1 is mostly nuclear [32], [56] (Fig. S4C, panel 2). Arsenite induces the localization of a fraction of CUGBP1 in SG [32], [56], and our immunofluorescence experiments showed that CUGBP1 was also recruited into bortezomib-induced SG (Fig. 3B). Quantification of these immunofluorescence data show that a significant fraction (∼45%) of total CUGBP1 is present in SG (Fig. 3B–C) where it co-localized with p21 mRNA (Fig. 3B and 3D–E) following treatment with bortezomib. Under these conditions, about 50% of CUGBP1 is nuclear (Fig. 3B–C) and only residual staining of CUGBP1 is detected in the cytoplasm (Fig. 3B and 3D). Prolonged treatment with bortezomib (10 h) resulted however in nuclear localization of most CUGBP1 (Fig. S4C). At this time we detected also some p21 mRNA FISH signal in the nucleus. However, and as mentioned above, the overall p21 mRNA FISH signal at 10-h bortezomib treatment is reduced (Fig. S4A and S4C), as compared to 4 h treatment (Fig. S4A; see also Fig. 2A) at which time point most of p21 mRNA (∼80%) co-localized with CUGBP1 in SG (Fig. 3B and 3D–E). Overall, our results (Fig. 3B) indicate that bortezomib treatment induces CUGBP1-p21 mRNA co-localization in SG. CUGBP1 might thus bind to p21 mRNA and recruit it to SG where it accumulates. The latter assumption would therefore predict that CUGBP1 depletion should trigger p21 mRNA degradation, which would explain the low amount of p21 protein following prolonged proteasome inhibition in CUGBP1-depleted cells (Fig. 3A). Indeed, we first confirmed that CUGBP1 binds to p21 mRNA under bortezomib conditions, as assessed by immunoprecipitating CUGBP1 (Fig. 3F) followed by quantitative RT-PCR (qRT-PCR) to detect associated p21 mRNA (Fig. 3G). This binding was specific, as suggested by the finding that control p27 mRNA was not recovered in CUGBP1 immunoprecipitates (Fig. 3G). Second, the steady-state p21 mRNA level significantly decreased in CUGBP1-depleted cells following proteasome inhibition (Fig. 3H). Depletion of CUGBP1 had however no effect on the steady-state level of p21 mRNA in untreated cells (Fig. S6A), suggesting that CUGBP1 is not required to maintain the basal level of the p21 mRNA. We then tested the possibility that the decreased level of p21 mRNA in CUGBP1-depleted cells upon bortezomib treatment might be consistent with a more rapid degradation of p21 mRNA. Indeed, the half-life of p21 mRNA in bortezomib conditions decreased from >3 h in mock-depleted cells (Fig. 4A) to 90 min ±15 min in CUGBP1-depleted cells (Fig. 4B). Depletion of CUGBP1 was efficient, as evidenced by both western blot analysis (Fig. 4C) and immunofluorescence (Fig. 4D), using anti-CUGBP1 antibodies. However, quantification of the localization of several SG markers (Fig. 4D, and data not shown) indicated that CUGBP1 depletion *per se* did not affect SG formation. This result ruled out the possibility that the destabilization of p21 mRNA observed in CUGBP1-depleted cells upon bortezomib treatment (Fig. 4A–B) was due to a cellular failure to form SG. One likely possibility of the destabilisation of p21 mRNA observed in CUGBP1-depleted cells is because it is no longer recruited to SG. We then assessed if CUGBP1 depletion affects the accumulation of p21 mRNA in SG using FISH. SG were visualized by immunofluorescence using antibodies to either Ras GTPase-activating protein-binding protein 1 G3BP1 (Fig. 4D) or FXR1 (data not shown). Whereas the major fraction of p21 mRNA (∼70%) is present in SG in over 60% of mock-depleted cells upon treatment with bortezomib (Fig. 4D–E), less than 30% of CUGBP1-depleted cells only exhibited such p21 mRNA localization in SG (Fig. 4D). Indeed, the large fraction of CUGBP1-depleted cells (∼80%) has a barely detectable FISH p21 mRNA signal in SG and in the cytoplasm (Fig. 4D and 4F). Under those bortezomib conditions, quantification of the intensity of p21 mRNA FISH signal showed that CUGBP1 depletion decreased this signal to 30–40%, as compared to mock-depleted cells where most p21 mRNA FISH signal was detected in SG (Fig. 4G). This effect seems to be specific since under the same conditions, depletion of CUGBP1 does not affect the association of the non-target GAPDH mRNA [47] with SG as assessed by immunofluorescence coupled with FISH (Fig. S7). Overall, our results show that CUGBP1 depletion significantly prevented bortezomib-induced p21 mRNA accumulation, which is likely due to its mistargeting in SG. As aforementioned, CUGBP1 depletion does not affect p21 mRNA steady-state levels in normal growth conditions (Fig. S6A) and we found that this is also the case under staurosporine treatment (Fig. S6B), a SG-free apoptotic condition [70] (Fig. S6D). These results suggest that CUGBP1 is not required to maintain the steady-state levels of p21 mRNA in the cytoplasm. On contrary, CUGBP1 seems to be required for bortezomib-induced p21 mRNA accumulation in SG (Fig. 3H, 4D, and S6C). We suggest that CUGBP1 promotes p21 mRNA accumulation by recruiting the transcript in SG, although we do not exclude that CUGBP1-mediated p21 mRNA accumulation involves additional mechanisms. Nevertheless, these results showed that CUGBP1 acts as a positive effector of p21 upregulation upon proteasome inhibition.

### p21 upregulation prevents bortezomib-mediated cell death

**Figure 5:**
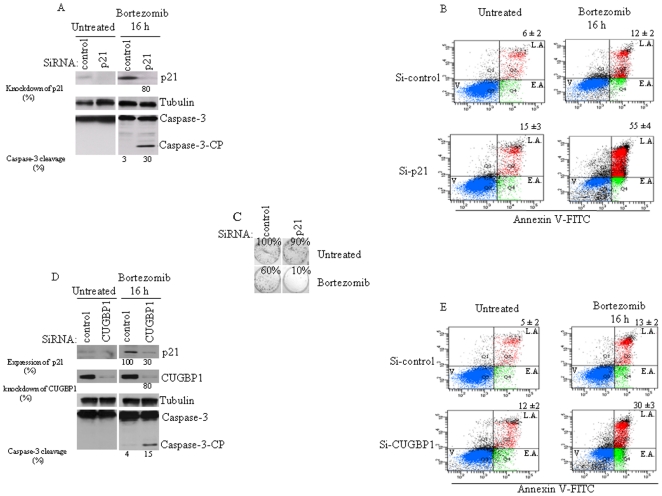
p21 depletion promotes bortezomib-mediated apoptosis. (A) HeLa cells were transfected with p21-directed siRNA, or with a control siRNA. Forty-eight hours later, cells were collected and their protein extracts analyzed by western blot for the amount of p21 (*top* panel) using anti-p21 antibodies. The activation of caspase-3 was analyzed using anti-caspase-3 antibodies (*bottom* panel). CP: cleaved product. Tubulin is used as a loading standard (*middle* panel). The amount of p21 was determined by quantitation of the signals on films by densitometry using Adobe Photoshop software and expressed as a percentage relative to the amounts of tubulin. The percentage of caspase-3 cleavage was calculated by quantifying the signal of the caspase-3 cleaved product relative to the amount of total caspase-3. Shown are representative results of three different experiments. (B) Following treatment with p21-specific siRNA or with a non-specific siRNA, HeLa cells were incubated with bortezomib for 16 h, then stained with annexin V-FITC and PI, and analyzed by flow cytometry. The percentage of total dead or dying cells (indicated at the *top* of each panel) was defined as the sum of early (*lower right* box) and late (*upper right* box) apoptosis and corresponds to the mean ± SEM from three independent experiments. V: viable cells; E.A: early apoptosis; L.A: late apoptosis. (C) Clonogenic assays. Following treatment with p21-specific siRNA or with a non-targeted siRNA, HeLa cells were incubated with bortezomib for 16 h, trypsinized, counted, replated in the absence of drug, and incubated for 10 d. Prior to counting colonies, cells were fixed, and then dried. Populations >50 cells were counted as one surviving colony. Data were calculated as the percentage of surviving colonies relative to the number found in control (untreated) plates. Results are expressed as the mean of triplicate measurements. (D–E) *Reducing CUGBP1 levels promotes bortezomib-induced apoptosis*. HeLa cells were treated with CUGBP1-specific siRNA, or with control siRNA. Seventy-two hours later, cells were either collected and their protein extracts analyzed by immunoblotting for the amount of p21, CUGBP1, tubulin (loading standard), and for caspase-3 activation, using corresponding antibodies (D), or stained with annexin V-FITC and PI, and analyzed by flow cytometry (E) as described above. In D, the relative expression of p21 and CUGBP1 was determined as described in Figure 3, and the percentage of caspase-3 cleavage was quantified as described in (A). Shown are typical results of three different experiments. In (C), the percentage of total dead or dying cells is indicated at the top of each panel in E and is defined as the sum of early (lower right box) and late (upper right box) apoptosis and represents the means +/− s.e.m, from two independent experiments.

Having revealed CUGBP1-mediated p21 mRNA accumulation as a pathway that promotes p21 upregulation following bortezomib treatment, we then investigated whether targeting this pathway might induce apoptosis. We have previously shown that suppressing SG formation induces massive apoptosis following bortezomib treatment [14]. We thus tested the effect of a decreased p21^WAF/CIP1^ expression on apoptosis upon bortezomib treatment. HeLa cells treated with p21-specific or control siRNAs were exposed to bortezomib (2 µM) for 16 h, and apoptosis was then measured (i) by western blot analysis of the cleavage of caspase-3, one of the main effectors of caspase-dependent apoptosis, as well as (ii) with the percentage of annexin V-positive cells detected using flow cytometry. Depletion of p21 ^WAF/CIP1^ was assessed by western blot (Fig. 5A) and did not induce significant apoptosis *per se* (Fig. 5A–B). Following a 16-h incubation with bortezomib, however, p21^WAF/CIP1^ depletion resulted in significant (30%) caspase-3 cleavage (Fig. 5A), while promoting apoptosis in a high percentage (55%) of cells (Fig. 5B). Similar results were obtained using a different p21-specific siRNA (data not shown). Moreover, this effect was not specific to HeLa cells since p21^WAF/CIP1^ depletion promoted bortezomib-induced apoptosis in Calu-I carcinoma cells (data not shown). Taken together, the present results thus strongly indicate that p21 upregulation can promote cancer cell resistance to bortezomib.

To further confirm the role of p21 in promoting resistance to bortezomib treatment, we performed clonogenic survival assays. HeLa cells were treated with p21-specific or control siRNAs, and following incubation with bortezomib, were washed to remove the drug, diluted and replated in fresh medium for 10 d, at which point colonies were counted. The results clearly showed that depleting p21^WAF/CIP1^ significantly (40%) decreased cell survival and proliferation following treatment with bortezomib (Fig. 5C). Overall, the present data suggest that resistance of cancer cells to bortezomib-induced apoptosis involves p21^WAF/CIP1^ upregulation.

### Depletion of CUGBP1 sensitizes HeLa cells to bortezomib-induced apoptosis

The role of CUGBP1 in cancer cell resistance to apoptosis has not been previously investigated. From the above-described results (Fig. 3–5), we hypothesized that CUGBP1 might promote resistance to bortezomib through p21 upregulation. In such a case, decreasing p21 expression via CUGBP1 depletion should promote apoptosis following bortezomib treatment. Thus, HeLa cells treated with CUGBP1-specific or control siRNAs were exposed to bortezomib (2 µM) for 16 h, and apoptosis was then assessed by caspase-3 cleavage (Fig. 5D) and annexin V assays (Fig. 5E). CUGBP1 depletion was confirmed by western blot (Fig. 5D) and did not induce significant apoptosis *per se* (Fig. 5D–E). Under bortezomib conditions, depletion of CUGBP1 induced cleavage of about 15% of total caspase-3 (Fig. 5D), and resulted in a significant percentage (30%) of cells undergoing apoptosis (Fig. 5E).

## Discussion

The present study clearly shows that bortezomib treatment of cancer cells upregulates p21^WAF1/CIP1^ expression, which confers cellular resistance to apoptosis. Bortezomib-mediated p21 upregulation involves p21 mRNA accumulation in SG and its stabilization through the SG component CUGBP1. Our results suggest the following two-step model for bortezomib-induced p21 upregulation. In the first step, p21 mRNA is quantitatively sequestered into SG in a CUGBP1-dependent fashion, thereby protecting the normally highly unstable p21 mRNA against degradation, and contributing to its accumulation. The second step involves SG disassembly, thus releasing the large pool of accumulated p21 mRNA which is now available for immediate translation. This creates a massive pulse of p21^WAF1/CIP1^ synthesis that helps preventing apoptosis despite proteasome inhibition by bortezomib. Our study therefore identified a potential survival pathway involving both CUGBP1 and p21 which could be therapeutically targeted to antagonize cancer cell resistance to bortezomib-mediated apoptosis.

Increasing evidence has implicated p21 as an anti-apoptotic factor that promotes cancer cell resistance to therapeutic agents. It has been shown that the treatment of cancer mesothelioma cells with chemotherapeutic drugs such as Irinotecan induces p21 expression and cellular senescence [71]. Depletion of p21 by specific short hairpin RNAs in tumor cells *in vivo* prevents Irinotecan-induced cell senescence, promotes apoptosis, and enhances the survival of Irinotecan–treated xenografts in animal models. p21 expression reduced the survival of Irinotecan–treated animals xenografted with mesothelioma tumor cells, in keeping with a potential role of p21 in chemoresistance [71]. Likewise, doxorubicin-treated breast, lung, and colon carcinoma cells undergo cellular senescence *in vitro*, which is characterized by a constitutively active ATM/ATR-dependent DNA damage response and the induction of p21 [72]. Blocking the ATM kinase with specific inhibitors suppresses p21 expression and induces apoptosis, suggesting that p21 acts downstream of ATM to promote cancer chemoresistance. It will be interesting to assess the hypothesis that chemotherapeutic drugs such as Irinotecan and doxorubicin can induce p21 upregulation through mechanisms involving the CUGBP1-SG pathway described in this work. A recent study has shown that imatinib, a BCR-ABL inhibitor used for the therapy of chronic myeloid leukemia (CML), downregulates p21 expression at both mRNA and protein levels in sensitive CML cells, while resistant CML cells exhibit increased p21 expression [73]. Overexpression of p21 mediated by transient transfection confers resistance to imatinib, a finding consistent with the hypothesis that p21 upregulation might promote resistance to chemotherapy. Our study shows that suppressing the expression of endogenous p21 is sufficient to prevent resistance to bortezomib-induced apoptosis. Future studies will be conducted to investigate the possibility that imatinib might prevent the bortezomib-mediated upregulation of p21 expression, which would sensitize cancer cells to apoptosis induction by the proteasome inhibitor.

The mechanisms by which p21 antagonizes apoptosis are not yet clear. p21 can block apoptosis indirectly by arresting cell cycle after nuclear binding to cyclin E-CDK2 and cyclin A-CDK2 complexes, or by increasing proliferation by promoting the nuclear accumulation of cyclin D-CDK4. However, FACS analysis of cell cycle progression shows that bortezomib does not affect cell cycle in our *in vitro* cancer cell model, despite the initial nuclear accumulation of p21 which is observed upon 10 h of bortezomib treatment. This result makes unlikely the possibility that p21-mediated resistance to bortezomib is due to its ability to arrest cell cycle. Moreover, protracted treatment (16 h) with bortezomib induces a cytoplasmic localization of p21. Stress-mediated cytoplasmic translocation of p21 involves its phosphorylation by kinases such as PKA and Akt [74], [75]. Cytoplasmic p21 has been shown to interact with the apoptotic effector caspase-3, in a PKA-phosphorylation dependant manner [76], and leading to resistance to Fas-mediated cell death [77], [78]. Alternatively, Akt-phosphorylated p21 can inhibit cell death induced by TNFα and other stimuli by inactivating ASK1 in the cytoplasm [37], [40], [75]. This MAPK kinase kinase activates the Jun N-terminal protein kinase in response to various genotoxic agents including proteasome inhibitors, which results with apoptosis [79]–[81]. Our immunoprecipitation experiments revealed an interaction between p21 and ASK1 during bortezomib treatment (Unpublished results). Although speculative at this stage, we propose that p21 inhibits bortezomib-mediated apoptosis by inactivating pro-apoptotic factors such as ASK1. Studies are underway to (i) determine the mechanisms underlying the cytoplasmic translocation of p21 and (ii) define if ASK1 is the target by which p21 prevents bortezomib-mediated apoptosis.

Our study also provides evidence that CUGBP1 might act as a potential factor in resistance to bortezomib-mediated apoptosis by promoting p21 mRNA stabilization/accumulation and expression. Previous studies have shown that CUGBP1 promotes p21 mRNA expression in normal cells by loading the transcript into translating ribosomes [54]. On contrary, other reports identified CUGBP1 as a factor that inhibits the expression of its target mRNAs by promoting their decay in the cytoplasm [47], [50], [52]. Our study identified a novel role of CUGBP1 in promoting p21 mRNA stabilization during proteasome inhibition, which could involve its recruitment to SG. First, qRT-PCR showed that bortezomib highly induces p21 mRNA stabilization leading to its accumulation. Second, FISH experiments revealed that the p21 mRNA thus accumulating is quantitatively recruited to bortezomib-induced SG. The possible contribution of SG in protecting p21 mRNA from turnover is supported by findings that bortezomib-induced p21 mRNA accumulation is lost in HRI-depleted cells, which do not form SG. Third, actinomycin D experiments show that CUGBP1 depletion prevents bortezomib-induced p21 mRNA stabilization. CUGBP1 depletion does not affect, however, SG formation. This exclude the possibility that p21 mRNA destabilization observed in CUGBP1-depleted cells upon bortezomib treatment is due to a failure to form SG. Quantification of FISH data show that CUGBP1 depletion significantly decreased the overall amount of p21 mRNA, as compared to mock-depleted cells where most of the accumulated p21 mRNA was detected in SG. These latter results further corroborate our qRT-PCR data showing that CUGBP1 is required for p21 mRNA accumulation upon bortezomib treatment. However, CUGBP1 depletion does not affect the steady-state levels of p21 mRNA under SG-free conditions tested, further supporting previous reports [47], [50], [52] which indicate that CUGBP1 may not act as a general stabilizing factor in the cytoplasm. We suggest that CUGBP1 promotes p21 mRNA stabilization upon bortezomib treatment indirectly by inducing its recruitment into SG. However, one cannot exclude the possibility that CUGBP1 is not essential for recruiting p21 mRNA to SG, but is required instead for sequestering p21 mRNA in these structures. In any case however, our results show that under bortezomib conditions, CUGBP1 is required for p21 mRNA segregation in SG thus promoting its stabilization. At this stage, the mechanism responsible for the stabilization of p21 mRNA in SG remains to be determined. In general, destabilization of the mRNA involves both removal of its 5′-end cap structure (or decapping) and deadenylation of its polyA tail, followed by exoribonuclease digestion from both ends. Proteins that protect either the cap structure or the polyA tail, such as eIF4E and PABP, respectively, have been identified as SG components [82], [83]. In contrast, enzymes that activate decapping or deadenylation, such as Dcp1a and PARN, respectively, are absent from SG [82], [84]. Thus, there is evidence that SG might protect p21 mRNA from degradation by isolating it from destabilizing factors, and CUGBP1 might promote p21 mRNA stabilization and accumulation either by recruitment or sequestration into SG. As a decaying factor, CUGBP1 was shown to interact directly with the PARN deadenylase to facilitate the removal of the poly(A) tail and thereby destabilize its target mRNAs [48]. It is thus tempting to speculate that the sequestration of CUGBP1 in SG prevented its association with PARN, thus protecting the associated p21 mRNA from turnover in SG. The association of CUGBP1 with p21 mRNA is likely to be subject to regulation by phosphorylation via the action of cyclin D3-cdk4/6 [85]. Interestingly, the residue targeted for phosphorylation by cyclinD3-cdk4/6 (Ser302) lies within the linker region of CUGBP1 [85], which has been shown to be required for localization in SG upon arsenite treatment [56]. Future investigations should determine if CUGBP1 phosphorylation at Ser302 is required for its localization in SG, as a prerequisite to p21 mRNA accumulation. Upon disassembly of SG, a large fraction of the p21 mRNA pool becomes available for rapid translation, thereby creating a transient pulse of high p21 expression. Previous studies have shown that CUGBP1 promotes p21 translation in senescent cells through binding to the GC-rich element in the 5′ region of p21 mRNA and loading of the mRNA onto ribosomes [54]. Following SG disassembly, therefore, CUGBP1 might also enhance p21 mRNA translation by promoting its association with ribosomes, although this remains to be demonstrated in a future study. In addition to GC-rich element, p21 mRNA contains two other regulatory elements, namely a AU-rich element and a GU-rich sequence located in its 3′-untranslated region. It is unlikely that the GU-rich element plays a role in the upregulation of p21 mRNA during proteasome inhibition, since this sequence is known to promote mRNA destabilization [49]. In contrast, the AU-rich element of p21 mRNA has been shown to confer p21 mRNA stabilization through its association with the RNA-binding protein HuR [43], [86]–[88]. However, our results show that HuR is dispensable for p21 mRNA accumulation during bortezomib treatment, suggesting that the AU-rich element is unlikely to be involved in the mechanism leading to p21 mRNA stabilization under bortezomib treatment. Studies are in progress to identify the cis-acting elements of p21 mRNA that interact with CUGBP1 in promoting localization and accumulation of this mRNA in SG. In addition to p21 mRNA, CUGBP1 might also promote localization of other mRNAs encoding antiapoptotic proteins in SG, thus leading to their accumulation. Several mRNAs encompassing GC-rich sequences in their 5′-region encode survival factors involved in the malignant phenotype, such as vascular endothelial growth factor (VEGF) and survivin [89], which could be potential targets of CUGBP1. Future studies will determine the role of CUGBP1 in the regulation of anti-apoptotic factors such as VEGF and survivin during chemotherapy.

### Conclusions

The present study has identified p21^WAF1/CIP1^ and its regulatory protein CUGBP1 as key players in the inhibition of apoptosis observed during treatment with bortezomib. Future studies should determine whether targeting these two factors might sensitize tumors to proteasome inhibitors in mouse models.

## Materials and Methods

### Cell Lines and Cultures

HeLa cervical cancer, Calu-1 lung cancer, and MCF-7 breast cancer cells were obtained from the American Type Culture Collection (Manassas, VA; ATCC). Cells were cultured in DMEM (Sigma, St. Louis, MO) supplemented with 10% fetal bovine serum (FBS; Sigma), penicillin, and streptomycin (Sigma). 293 cells stably transfected with green fluorescent protein ubiquitin (GFPu) were purchased from ATCC and handled as previously described [57], [58].

### Drugs and Drug Treatments

Bortezomib was purchased from LC Laboratories (Woburn, MA) and dissolved in DMSO as a 65 mM stock solution, aliquoted and stored at −20°C. Bortezomib treatment was performed when cells had reached 60–80% confluence. Staurosporine was purchased from Sigma.

### Antibodies

Phospho-specific anti-eIF2α and the pan anti-eIF2 were purchased from Cell Signaling Technology (Beverly, MA). Anti-HuR, anti-G3BP1, anti-FMRP, anti-FXR1, and anti-eIF4E were made in-house and have been previously described [14], [76]. Anti-CUGBP1, anti-p21, and anti-HRI antibodies were purchased from Santa Cruz Biotech (Santa Cruz, CA). The anti-tubulin and anti-caspase-3 antibodies were obtained from Developmental Studies Hybridoma Bank (Iowa City, IA), and Cell Signaling (Danvers, MA), respectively. Anti-p27 and anti-GFP antibodies were purchased from BD Bioscience and Abcam, respectively.

### Small-Interfering RNA (siRNA) Experiments

siRNA-p21 were purchased as validated siRNAs from Qiagen (Mississauga, ON, Canada). siRNA-CUGBP1, siRNA-HuR, siRNA-HRI, and siRNA non-targeting control were obtained from Dharmacon (Lafayette, CO). siRNA transfections were performed essentially as described [14], [76], using Hiperfect reagent (Qiagen) following the manufacturer's protocol. Twenty-four hours before transfection, cells were trypsinized and plated at a density allowing to reach 60–80% confluence after 24 h. For a 6-well plate, annealed duplexes were used at a final concentration of 5 nM. Seventy-two hours post-transfection, cells were either fixed and processed for immunofluorescence, or harvested for protein extraction.

siRNA-p21: 5′ AAGACCATGTGGACCTGTCAC 3′

siRNA-CUGBP1: 5′-GAGCCGAGGTTGTGCATTT-3′

siRNA-HuR: 5′-GGGATAAAGTAGCAGGACA-3′

si-HRI: 5′-GATCTGAAGTGGAAGCTAA-3′

### Immunofluorescence and RNA FISH

Following fixation and permeabilization (20 minutes in 3.7% paraformaldehyde at room temperature followed by a 15-min immersion in MeOH at −20°C), fixed cells were incubated with primary antibodies diluted in 0.1% Tween-20/PBS (PBST) for 2 h at room temperature. After washing with PBST, cells were incubated with goat anti-mouse/rabbit IgG (H+L) secondary antibodies conjugated with the Alexa Fluor dye with the appropriate absorption maximum (405/488/594) for 1 h, washed, and then mounted.

For FISH experiments, a DNA fragment encompassing the p21 coding region was amplified by PCR using primers fused either with T3 (p21-forward: 5′- AATTAACCCTCACTAAAGGGATGTCCGTCAGAACCCATGC-3′) or T7 (p21-reverse: 5′-TAATACGACTCACTATAGGGGTTAGGGCTTCCTCTTGGAGA-3′) minimal promoter sequences. The DNA fragment encompassing the GAPDH coding region was amplified by PCR using primers fused either to the T3 (GAPDH-forward: 5′- AATTAACCCTCACTAAAGGGAAACTGTGGCGTGATG-3′) or T7 (GAPDH-reverse: 5′-TAATACGACTCACTATAGGGTTACTCCTTGGAGGCCATG-3′) minimal promoter sequences. The amplified fragments were used as templates for *in vitro* transcription to produce either a p21 or GAPDH antisense RNAs from the T7 promoter, or p21 and GAPDH sense RNAs from the T3 promoter, using the FISH Tag RNA Green Kit with Alexa Fluor 488 (Invitrogen, Burlington, ON, Canada). In the latter technique, *in vitro* transcription incorporates an amine-modified UTP into the probe template. The purified RNA is then incubated with an amine-reactive Alexa Fluor 488 dye (e.g. the succinimidyl ester of Alexa Fluor 488 carboxylic acid) which binds and reacts with the modified UTP. The conjugated probe is then purified, quantified, denatured, and incubated with cells. Before hybridization, cells were fixed and permeabilized as described above, and then prehybridized in 50% PBST/50% hybridization buffer (50% formamide, 5X SSC, 1 mM phosphate buffer, pH 7.4, 1X Denhardt's solution, and 160 ng/µl of denatured salmon sperm DNA) at room temperature for 10 min with gentle rocking. After two washes with fresh hybridization buffer for 30 min at 42°C, the probe was added to the hybridization buffer and incubated with the cells for 16 h at 42°C. After hybridization, cells were processed for immunofluorescence as described above. Probes were visualized using the LSM 700 laser scanning confocal Axio Observer. Z1 microscope (Zeiss), equipped with a software ZEN 2009 for image acquisition and analysis. Images were acquired through the following settings: 63X oil objective (zoom 1.0), 0.06 um for pixel size, and 1.00 airy units (AU) as Pinhole.

### Immunoprecipitation, Quantitative RT-PCR and Actinomycin D Experiments

For immunoprecipitation, cells were collected and lysed at 4°C with a lysis buffer (50 mM Tris-HCl, pH 7.4; 0.5% NP-40; 150 mM NaCl; 1 mM MgCl_2_; 0.25 mM phenylmethanesulfonylfluoride; 0.5 mM DTT) containing a cocktail of protease inhibitors (Roche, Laval, QC, Canada) and 40 U/µl RNase Inhibitor (Invitrogen). The extract was then incubated with protein A Sepharose CL-4B beads (GE Healthcare Life Sciences, Baie-d'Urfé, QC, Canada) conjugated with the appropriate antibody. Following three washes with lysis buffer, the beads were resuspended with an equal volume of washing buffer. Five percent of the suspension was used for immunoblot analysis of the immunoprecipitated proteins, and the remainder was used for RNA isolation. Briefly, following digestion with proteinase K, RNA was extracted with phenol/CHCl_3_ and precipitated with isopropanol in the presence of glycogen. RNA was resuspended in water and analyzed by qRT-PCR.

RT-PCR reactions were performed using the Quantitect Reverse Transcriptase kit (Qiagen). Each reaction contained 2 µl of RNA (isolated using the RNeasy Plus Mini Kit; Qiagen) at 100 ng/µl, 10 µl of RNase-free water, 2 µl of genomic DNA Wipeout Buffer 7X, 4 µl of Quantiscript RT Buffer 5X, 1 µl of RT Primer Mix and 1 µl of Quantiscript Reverse Transcriptase.

Real-time PCR reactions were prepared using the Power SYBR® Green PCR Master mix (Applied Biosystems, Streetsville, ON, Canada) in a total volume of 25 µl: 12.5 µl of PCR Master Mix, 0.67 µl of forward primer at 3.75 µM, 0.67 µl of reverse primer at 3.75 µM, 9.2 µl of deionized (Milli-Q grade) water and 2 µl of RT-PCR. Reactions were run and data then analyzed using the MX3000 Real-Time PCR system (Applied Biosystems) with a 4-stage program: (1) 2 min at 50°C, (2) 10 min at 95°C, (3) 40 cycles of a 2-step reaction: 95°C ×15 s and 55°C ×60 s, and (4) a 3-step reaction: 95°C ×15 s, 60°C ×20 s and 95°C ×15 s.

To prepare templates for the p21 mRNA, the oligonucleotide pair used was: 5′-GACTTTGTCACCGAGACACC-3′ (forward), and 5′-GACAGGTCCACATGGTCTTC-3′ (reverse). For preparing templates corresponding to the p27 mRNA, the oligonucleotide pair used was: 5′-AAGAGTTAACCCGGGACTTG-3′ (forward), and 5′-CCACTCGTACTTGCCCTCTA-3′ (reverse). For preparing templates corresponding to the GAPDH mRNA, the oligonucleotide pair used was: 5′-ACGACCACTTTGTCAAGCTC-3′ (forward), and 5′-GTTGCTGTAGCCAAATTCGT-3′ (reverse).

For actinomycin D experiments, transcription was blocked using 5 µg/mL actinomycin D. RNA was isolated and analyzed as described above.

### Annexin V-Fluorescein Isothiocyanate/Propidium Iodide Assay and FACS Analysis

At the end of the experimental treatment, both adherent and detached cells were harvested. Cells were washed with ice-cold PBS, then pelleted again at 1500 rpm for 10 min at 4°C, and resuspended in ice-cold binding buffer (10 mM HEPES/NaOH, pH 7.4, 140 mM NaCl, 2.5 mM CaCl_2_). Cells were subsequently stained with annexin V-fluorescein isothiocyanate (FITC) and propidium iodide (PI) for 15 min in the dark. A total of 5×10^4^ cells were counted, and dead cells were examined by flow cytometry. For FACS analysis, collected cells were fixed with 3% paraformaldehyde, washed with PBS, and stained with 4′,6-diamidino-2-phenylindole (DAPI) (1 µg/mL) and analyzed by flow cytometry.

### Clonogenic Survival Assay

Cells were plated in duplicate and incubated for 24 h. Following treatment, cells were washed with PBS, trypsinized, counted, replated in 6-well plates at 10^3^ cells/well in the absence of drug, and incubated for 10 d. Before colony counting, cells were washed with PBS, stained (0.1% (w/v) crystal violet in a 0.0037% (v/v) formaldehyde solution in PBS), rinsed with deionized H_2_O, and dried. Populations >50 cells were counted as one surviving colony.

## Supporting Information

Figure S1 **Time course analysis of SG formation and p21 expression during bortezomib treatment.** (A) HeLa cells were treated with 2 µM bortezomib for the indicated period and then processed for immunofluorescence. SG were visualized with anti-FMRP antibodies and p21 protein was detected using anti-p21 antibodies. The percentage of cells harboring SG from five different fields and three different experiments containing a total of 1000 cells is indicated on the bottom of each merge pictures. (B) HeLa cells were treated with 2 µM bortezomib for the indicated period then collected for western blot analysis. Proteins were extracted and analyzed for the expression of p21 and as a loading control G3BP1, using the corresponding antibodies. The amount of p21 was determined by quantitation of the signals on films by densitometry using Adobe Photoshop software and expressed as percentage relative to the amounts of G3BP1. (C) qRT-PCR of p21 mRNA. Following treatment with 2 µM bortezomib for the indicated period, cells were collected and total RNA content was then isolated. The amount of p21 was quantified by real time-PCR relative to GAPDH mRNA using the ΔΔCt method. Results are expressed as the mean ± SEM (error bars) of triplicate measurements. (D-E) Quantification of p21 immunofluorescence signal in nuclei versus the cytoplasm of cells treated with bortezomib for either, 10 h (D) or 16 h (E), using Adobe Photoshop software.
(TIF)

Figure S2 **Bortezomib upregulates p21 mRNA expression in different cancer cells.** (A-B) Calu-I and MCF-7 cells were treated with bortezomib and processed for immunofluorescence (A), or lysed, and their protein extracts were analyzed for the levels of p21 and tubulin (B), as described in the legend to Figure 1.
(TIF)

Figure S3 **Expression profile of proteasome substrates during bortezomib treatment.** GFPu-expressing 293 cells (A) and HeLa (B) were treated with 2 µM bortezomib for the indicated time. Proteins were extracted and analyzed for the expression of GFPu (A) and p27 (B), using the corresponding antibodies. G3BP1 was used as a loading control. The amounts of GFPu and p27 were determined by quantitation of the signals on films by densitometry using Adobe Photoshop software and expressed as a percentage relative to the amounts of G3BP1.
(TIF)

Figure S4 **Prolonged treatment with bortezomib induces a redistribution of the p21 mRNA in the cytoplasm and nuclei.** (A-B) HeLa cells were treated with 2 µM bortezomib for the indicated periods. SG were visualized with anti-FMRP and anti-G3BP1 antibodies, and p21 mRNA was detected by FISH. The percentage of SG is indicated. The percentage of cells harboring SG positive for p21 mRNA is also indicated. Shown are typical results from five different fields and three different experiments containing a total of more than 1000 cells. (B) Densitometry quantification of p21 mRNA FISH signal in SG versus the cytoplasm was done with Adobe Photoshop software as described in Figure 2B. (C) HeLa cells were treated with 2 µM bortezomib for 10 h. SG were visualized with anti-CUGBP1 antibodies. DAPI depicts nuclei.
(TIF)

Figure S5 **HRI depletion prevents bortezomib-induced SG formation and p21 mRNA accumulation.** (A-C) HeLa cells were treated with HRI-specific siRNA or with control siRNA, and then incubated with bortezomib (2 µM) for 4 h. (A-B) Cells were harvested and their RNA and proteins content isolated. (A) Levels of p21 mRNA was measured by qRT-PCR and standardized against GAPDH mRNA as described in the legend of Figure 2. (B) Depletion of HRI was assessed by western blot using anti-HRI antibodies. (C) Cells were processed for immunofluorescence to detect SG using anti-G3BP and anti-FMRP antibodies. The percentage of cells harboring SG from five different fields and three different experiments containing a total of 1000 cells is indicated.
(TIF)

Figure S6 **CUGBP1 depletion does not affect the steady-state level of the p21 mRNA in SG-free conditions tested.** (A-C) Seventy-two hours following transfection with CUGBP1, or control siRNAs, cells were left untreated (A), or were treated with 1 µM staurosporine (B) or 2 µM bortezomib (C) for 4 h, as indicated. Total RNA was isolated and the level of p21 mRNA expression was determined by qRT-PCR, standardized against GAPDH mRNA, and quantified as described in Figure 2. Results are expressed as a percentage of the mRNA levels present in mock-depleted cells. (D) Staurosporine treatment does not induce SG. HeLa cells were treated with bortezomib (2 µM, 4 h) or Staurosporine (1 µM, 4 h) then processed to visualize SG using anti-FMRP and anti-G3BP1 antibodies as described above. DAPI depicts nuclei. The percentage of SG is indicated at the top of each panel.
(TIF)

Figure S7 **CUGBP1 depletion does not affect GAPDH mRNA localization in SG.** HeLa cells were treated with non-specific, or with CUGBP1-directed siRNA, incubated with bortezomib (2 µM) for 4 h and processed for FISH to detect GAPDH mRNA coupled with immunofluorescence to visualize SG using antibodies against CUGBP1 and G3BP1 proteins. Immunofluorescence using anti-CUGBP1 antibodies is used to monitor CUGBP1 depletion. The percentage of SG is indicated at the bottom of the right panels. The percentage of cells harboring SG positives for GAPDH mRNA is also indicated. Those percentages are representative of typical results from three different experiments counting more than 1000 cells.
(TIF)
