# Supplementary figures and images for: p21WAF1/CIP1 Upregulation through the Stress Granule-Associated Protein CUGBP1 Confers Resistance to Bortezomib-Mediated Apoptosis
Source: PLoS One. 2011 May 26;6(5):e20254. doi: 10.1371/journal.pone.0020254 (PMC3102688; doi:10.1371/journal.pone.0020254)

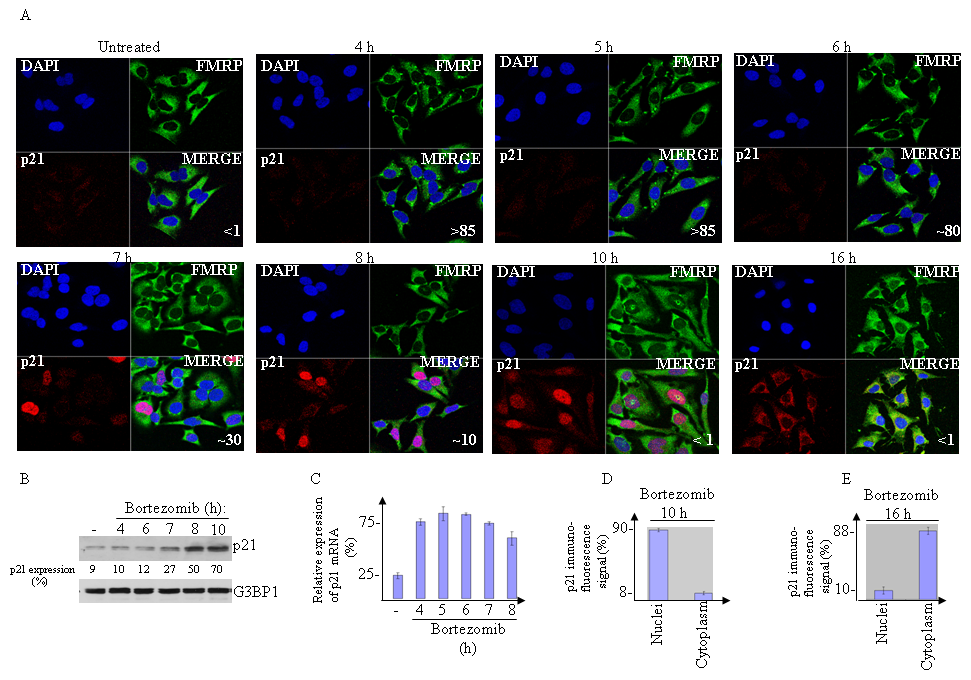

Supplement: Figure S1 — Time course analysis of SG formation and p21 expression during bortezomib treatment. (A) HeLa cells were treated with 2 µM bortezomib for the indicated period and then processed for immunofluorescence. SG were visualized with anti-FMRP antibodies and p21 protein was detected using anti-p21 antibodies. The percentage of cells harboring SG from five different fields and three different experiments containing a total of 1000 cells is indicated on the bottom of each merge pictures. (B) HeLa cells were treated with 2 µM bortezomib for the indicated period then collected for western blot analysis. Proteins were extracted and analyzed for the expression of p21 and as a loading control G3BP1, using the corresponding antibodies. The amount of p21 was determined by quantitation of the signals on films by densitometry using Adobe Photoshop software and expressed as percentage relative to the amounts of G3BP1. (C) qRT-PCR of p21 mRNA. Following treatment with 2 µM bortezomib for the indicated period, cells were collected and total RNA content was then isolated. The amount of p21 was quantified by real time-PCR relative to GAPDH mRNA using the ΔΔCt method. Results are expressed as the mean ± SEM (error bars) of triplicate measurements. (D-E) Quantification of p21 immunofluorescence signal in nuclei versus the cytoplasm of cells treated with bortezomib for either, 10 h (D) or 16 h (E), using Adobe Photoshop software. (TIF) [file pone.0020254.s001.tif]

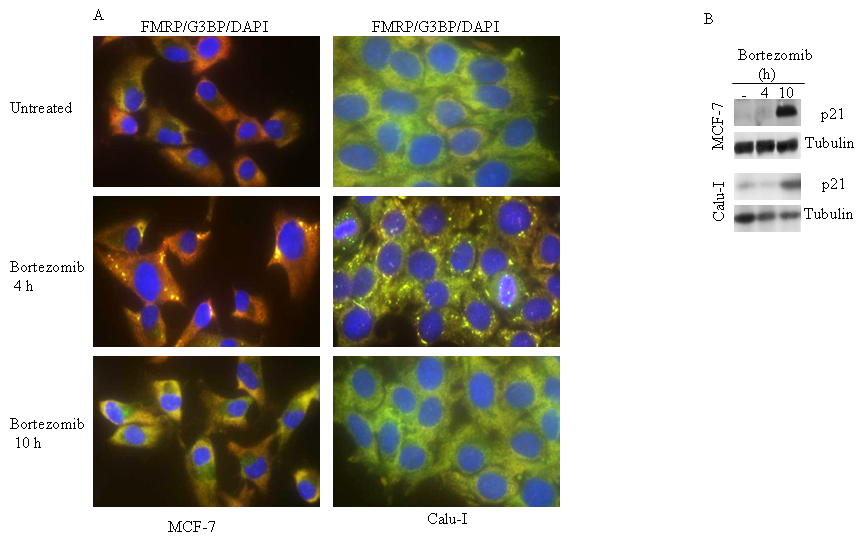

Supplement: Figure S2 — Bortezomib upregulates p21 mRNA expression in different cancer cells. (A-B) Calu-I and MCF-7 cells were treated with bortezomib and processed for immunofluorescence (A), or lysed, and their protein extracts were analyzed for the levels of p21 and tubulin (B), as described in the legend to Figure 1. (TIF) [file pone.0020254.s002.tif]

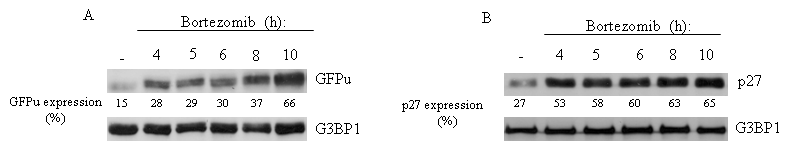

Supplement: Figure S3 — Expression profile of proteasome substrates during bortezomib treatment. GFPu-expressing 293 cells (A) and HeLa (B) were treated with 2 µM bortezomib for the indicated time. Proteins were extracted and analyzed for the expression of GFPu (A) and p27 (B), using the corresponding antibodies. G3BP1 was used as a loading control. The amounts of GFPu and p27 were determined by quantitation of the signals on films by densitometry using Adobe Photoshop software and expressed as a percentage relative to the amounts of G3BP1. (TIF) [file pone.0020254.s003.tif]

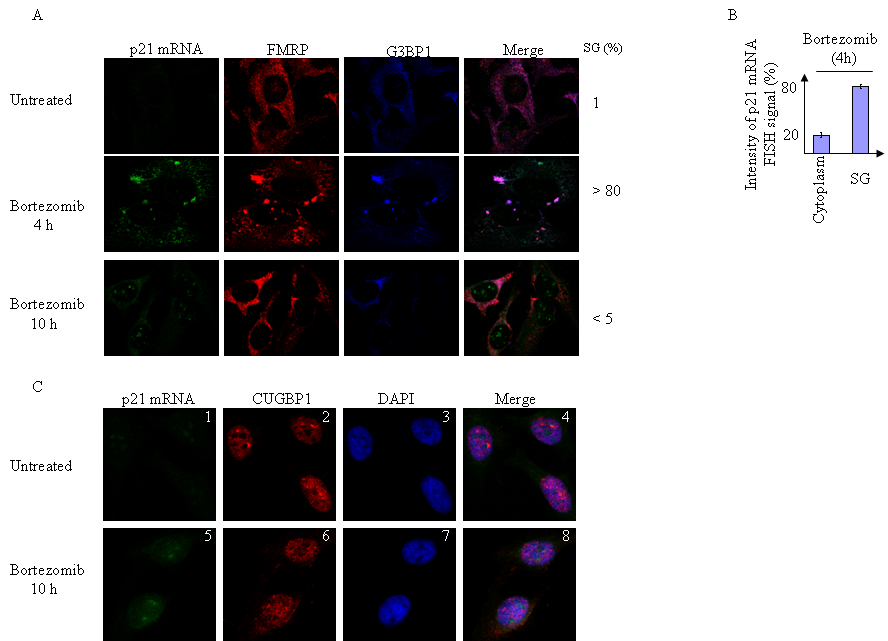

Supplement: Figure S4 — Prolonged treatment with bortezomib induces a redistribution of the p21 mRNA in the cytoplasm and nuclei. (A-B) HeLa cells were treated with 2 µM bortezomib for the indicated periods. SG were visualized with anti-FMRP and anti-G3BP1 antibodies, and p21 mRNA was detected by FISH. The percentage of SG is indicated. The percentage of cells harboring SG positive for p21 mRNA is also indicated. Shown are typical results from five different fields and three different experiments containing a total of more than 1000 cells. (B) Densitometry quantification of p21 mRNA FISH signal in SG versus the cytoplasm was done with Adobe Photoshop software as described in Figure 2B. (C) HeLa cells were treated with 2 µM bortezomib for 10 h. SG were visualized with anti-CUGBP1 antibodies. DAPI depicts nuclei. (TIF) [file pone.0020254.s004.tif]

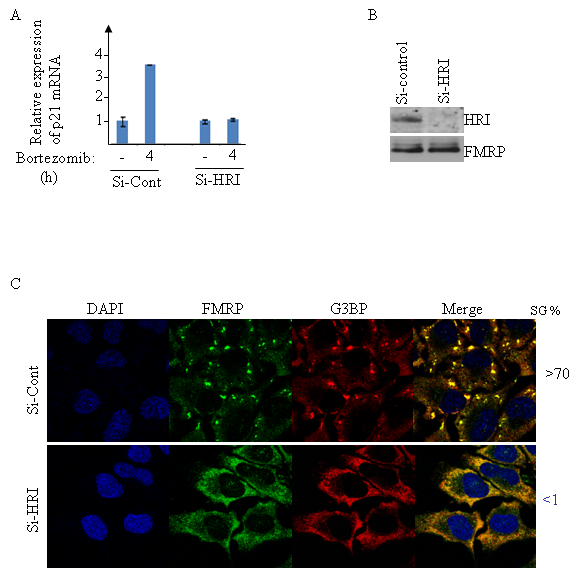

Supplement: Figure S5 — HRI depletion prevents bortezomib-induced SG formation and p21 mRNA accumulation. (A-C) HeLa cells were treated with HRI-specific siRNA or with control siRNA, and then incubated with bortezomib (2 µM) for 4 h. (A-B) Cells were harvested and their RNA and proteins content isolated. (A) Levels of p21 mRNA was measured by qRT-PCR and standardized against GAPDH mRNA as described in the legend of Figure 2. (B) Depletion of HRI was assessed by western blot using anti-HRI antibodies. (C) Cells were processed for immunofluorescence to detect SG using anti-G3BP and anti-FMRP antibodies. The percentage of cells harboring SG from five different fields and three different experiments containing a total of 1000 cells is indicated. (TIF) [file pone.0020254.s005.tif]

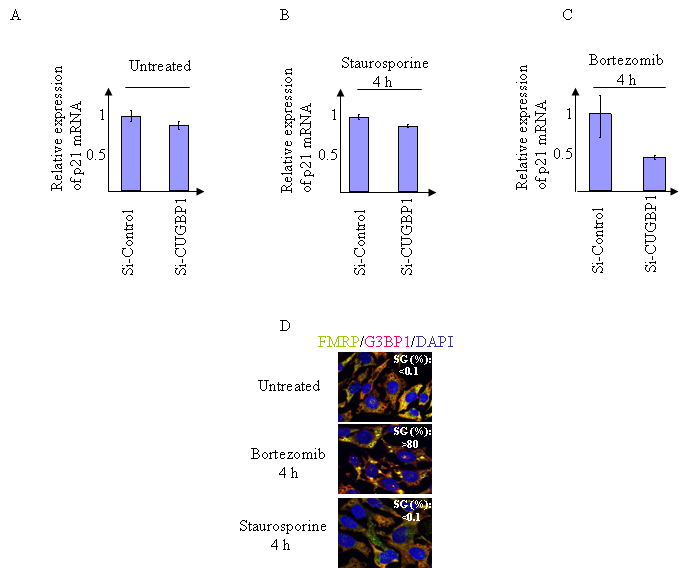

Supplement: Figure S6 — CUGBP1 depletion does not affect the steady-state level of the p21 mRNA in SG-free conditions tested. (A-C) Seventy-two hours following transfection with CUGBP1, or control siRNAs, cells were left untreated (A), or were treated with 1 µM staurosporine (B) or 2 µM bortezomib (C) for 4 h, as indicated. Total RNA was isolated and the level of p21 mRNA expression was determined by qRT-PCR, standardized against GAPDH mRNA, and quantified as described in Figure 2. Results are expressed as a percentage of the mRNA levels present in mock-depleted cells. (D) Staurosporine treatment does not induce SG. HeLa cells were treated with bortezomib (2 µM, 4 h) or Staurosporine (1 µM, 4 h) then processed to visualize SG using anti-FMRP and anti-G3BP1 antibodies as described above. DAPI depicts nuclei. The percentage of SG is indicated at the top of each panel. (TIF) [file pone.0020254.s006.tif]

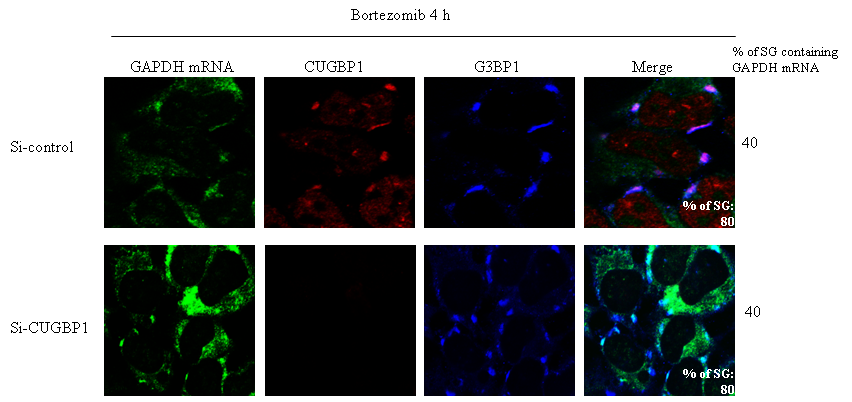

Supplement: Figure S7 — CUGBP1 depletion does not affect GAPDH mRNA localization in SG. HeLa cells were treated with non-specific, or with CUGBP1-directed siRNA, incubated with bortezomib (2 µM) for 4 h and processed for FISH to detect GAPDH mRNA coupled with immunofluorescence to visualize SG using antibodies against CUGBP1 and G3BP1 proteins. Immunofluorescence using anti-CUGBP1 antibodies is used to monitor CUGBP1 depletion. The percentage of SG is indicated at the bottom of the right panels. The percentage of cells harboring SG positives for GAPDH mRNA is also indicated. Those percentages are representative of typical results from three different experiments counting more than 1000 cells. (TIF) [file pone.0020254.s007.tif]
